# Fibroin nanoparticles: a promising drug delivery system

**DOI:** 10.1080/10717544.2020.1736208

**Published:** 2020-03-11

**Authors:** Duy Toan Pham, Waree Tiyaboonchai

**Affiliations:** a Faculty of Pharmaceutical Sciences, Naresuan University, Phitsanulok, Thailand;; b Department of Chemistry and Center of Excellence for Innovation in Chemistry, Faculty of Science, The Center of Excellence for Innovation in Chemistry (PERCH-CIC), Mahidol University, Salaya, Thailand

**Keywords:** Fibroin, nanoparticles, biomaterial, review, administrative route

## Abstract

Fibroin is a dominant silk protein that possesses ideal properties as a biomaterial for drug delivery. Recently, the development of fibroin nanoparticles (FNPs) for various biomedical applications has been extensively studied. Due to their versatility and chemical modifiability, FNPs can encapsulate different types of therapeutic compounds, including small and big molecules, proteins, enzymes, vaccines, and genetic materials. Moreover, FNPs are able to be administered both parenterally and non-parenterally. This review summaries basic information on the silk and fibroin origin and characteristics, followed by the up-to-date data on the FNPs preparation and characterization methods. In addition, their medical applications as a drug delivery system are in-depth explored based on several administrative routes of parenteral, oral, transdermal, ocular, orthopedic, and respiratory. Finally, the challenges and suggested solutions, as well as the future outlooks of these systems are discussed.

## 1. Introduction

In the modern era of personalized medicine, the effectiveness of a drug is not only based on its therapeutic efficacy but also, more importantly, its unwanted effects. These side effects, which vary individually from mild to severe, are often the main factor limiting a drug usage. Most, if not all, unwanted effects result from the vast bodily distribution of pharmaceutical agents. For conventional dosage forms (i.e. tablets, capsules, intravenous injections), only a small fraction of the administered dose reaches the target site, while a majority of the drug molecules distributes all over the entire body based on its physicochemical properties. For instance, less than 0.5% of a paclitaxel intravenous injection initial dose is available locally within the lung tumor to exert its therapeutic effect (Sparreboom et al., 1996). Thus, to overcome this drawback, it is crucial to develop a drug delivery system that transports the drug directly to the target sites, consequently optimizing its action while reducing its toxic side effects.

First proposed by Paul Ehrlich more than a hundred years ago, the tiny submicron ‘magic bullet’ was described as ‘drugs that go straight to their intended cell-structural targets’, without targeting any unwanted tissues (Strebhardt & Ullrich, 2008). One of the most commonly investigated ‘magic bullet’ is nanoparticles (NPs). By entrapping, encapsulating, adsorbing, or chemically binding the drug molecules, these 1–1000-nm-in-diameter particles provide numerous clinical benefits. They can (1) protect drugs from degradation, (2) solubilize and/or enhance their permeability, (3) control the drug release profiles, (4) alter their pharmacokinetics, (5) selectively increase cellular uptake, and (6) target the disease site specifically (Date et al., 2016; Pham et al., 2018a, 2019a). Typically, NPs are categorized into three types, including inorganic (i.e. gold NPs), lipid-based (i.e. liposomes), and polymer-based (i.e. protein NPs). In the US market, FDA has approved more than 100 nanopharmaceutical products, with 46 inorganic NPs, 21 lipid-based NPs, and 11 polymer-based NPs (Etheridge et al., 2013). Among them, the inorganic NPs are the easiest one to fabricate; however, their biggest drawback is non-biodegradable property that causes long-term toxicity to the human biological system and the environment (Matteis, 2017). On the other hand, the lipid-based NPs are biocompatible, but possess drawbacks of their complex ingredients (i.e. surfactant, solid lipid, liquid lipid), formulating processes, and stability (Mehnert & Mäder, 2001). The polymer-based NPs, which have both biocompatibility and stability profile, therefore, have gained increasingly interests.

Utilizing polymers as a carrier, both synthetic and natural, polymer-based NPs can encapsulate the drug entity into its core (nanocapsules) or distribute the drug evenly in its matrix (nanospheres) (Soppimath et al., 2001; Couvreur et al., 2002). Synthetic polymers such as polyesters, polyanhydrides, and polyphosphazenes have been used extensively (Nair & Laurencin, 2006; Liechty et al., 2010). Due to their bio-incompatibility, only a handful of candidates have been approved by the FDA, such as poly(lactic-co-glycolic acid) (PLGA) (Mao et al., 2012). Nevertheless, the PLGA NPs degradation products (i.e. lactic acid) decrease the pH locally, which consequently denature the entrapped drugs, especially acid-labile proteins, and reduce their therapeutic effects (Estey et al., 2006; Giteau et al., 2008). Hence, various natural polymers, namely chitosan (Tiyaboonchai, 2003; Mohammed et al., 2017; Naskar et al., 2019), alginates (Sosnik, 2014), zein (Pascoli et al., 2018), gelatin (Sahoo et al., 2015; Yasmin et al., 2017), and fibroin (Mathur & Gupta, 2010; Aramwit, 2012; Zhao et al., 2015; Gianak et al., 2019) have been employed as alternatives with high biocompatibility and biodegradability. Among them, fibroin is a FDA-approved polymer that has been popularly used in numerous medical applications such as sutures, tissue regeneration, coating devices, and drug delivery systems (Altman et al., 2003; Numata & Kaplan, 2010). Mostly extracted from the cocoons of domesticated silkworms *Bombyx mori*, fibroin have gained increasingly interests due to its excellent mechanical properties and high biocompatibility, biodegradability, inexpensiveness, and preparation flexibility (Pham et al., 2018a, 2019a). These characteristics are ideal for fibroin nanoparticles (FNPs) formulation. Moreover, due to its amphoteric properties, fibroin can be further strengthened and modified by crosslinking by itself or with other positively charged polymers such as poly(ethylenimine) (PEI) (Pham et al., 2018a). In the last two decades, numerous FNPs publications for drug delivery systems have been continuously reported. Therefore, in this review, we summarized the basic information on the silk and fibroin origin and characteristics, followed by the up-to-date data on the FNPs preparation and characterization methods, and their medical applications as a drug delivery system.

## 2. Silk origin and fibroin extraction

### 2.1. Silk origin

It is worth to clarify that more than 30,000 spider species and approximately 113,000 species in the insect order Lepidoptera, as well as numerous other insect orders, can produce thousands different kinds of silks (Kaplan et al., 1993). Many of them are still uncharacterized. Among them, more than 90% of the silk supply is mulberry silk, which is produced mostly by the silkworm *Bombyx mori*.

Of more than 5000-year history of textile industry, silk has been widely used all over the world, especially in the Asia countries. The silk earliest evidence was a *Bombyx mori* silk cocoon found in Xia County, Shanxi, early China, which was carbon-dated back to between 4000 and 3000 BC (Vainker, 2004). Similarly, the first silk fabric, originated from 3630 BC, in Henan (the birthplace of Chinese civilization), was used to wrap a child body (Vainker, 2004). For some thousands years, China was the only country fabricating silk, as the technique was a guarded top-notch secret within the Chinese empires. Traders to neighbor countries clarified silk as a material ‘derived from the wool of sheep sprinkled with water and exposed to sunshine’ (Holland et al., 2019). The secret was later lost to other Asian countries such as Korea, Japan, and India. The Western cultures first acknowledged silk from approximately the second century BC, when the Han dynasty in China (206 BC–220 AD) established the Silk Road, a commercial network connecting the East (Chang’an, now Xi’an, China) and the West (Mediterranean Sea, Roman Empire) (Belanger, 2011). Although various products and knowledge were exchanged, including paper, gunpowder, and religions such as Buddhism, silk remained the major trade item exported from China (Belanger, 2011).

### 2.2. Fibroin extraction

Generally, fibroin can be extracted from the silkworm cocoons. A silk fiber composes of fibroin (∼75% w/w) as a core and sericin (∼25% w/w), a glue-like protein, wrapped around fibroin as the outer layer (Kaplan et al., 1993). Sericin composes of a series of water-soluble globular glycoproteins that can cause immunological responses (Aramwit, 2012; Melke et al., 2016), and can be removed by a thermo-chemical process called degumming (Pham et al., 2018b). Unethically, without the need of degumming, fibroin can be extracted directly from the worm posterior glands by dissecting the mature fifth instar silkworm larvae (Mandal & Kundu, 2008). In this case, fibroin is in liquid water soluble form and is regarded as silk I. On the other hand, the degummed silk fiber consists of insoluble fibroin (silk II), and thus, requires further treatment to be transformed back to silk I. The product of this process is commonly called regenerated fibroin.

**Figure 1.**
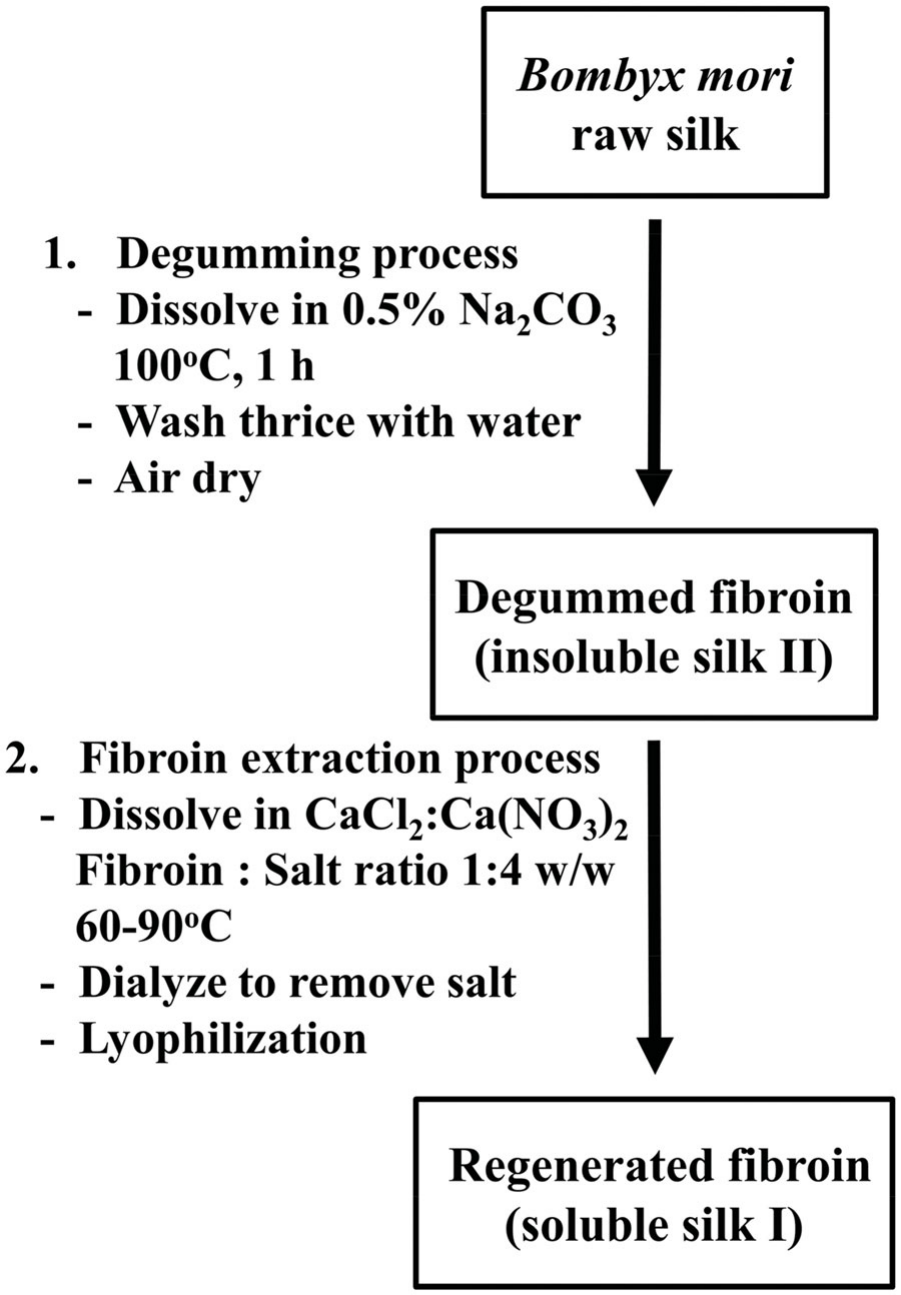
Silk fibroin degumming and extracting process.

A typical protocol for producing regenerated fibroin from the silkworm cocoons is presented in Figure 1 (Rockwood et al., 2011; Pham et al., 2018a, 2019a). The methods might be slightly different from several studies, however, they all utilize Na_2_CO_3_ as a degumming agent and a chaotropic salt solution as a silk II-to-silk I transformating agent. For instance, cocoon small-sized pieces are dissolved in Na_2_CO_3_ solution (degumming step), followed by boiling at 100 °C for 30–60 min. The silk fibroin, which is insoluble in Na_2_CO_3_, will be washed thrice with ultrapure water, air dried, and can be stored at room temperature. To make regenerated fibroin, the fibroin from previous step is further dissolved in LiBr or CaCl_2_ (fibroin:salt solution ratio of 1:4 w/w), followed by heating to 60–90 °C. The viscous liquid is then dialyzed against ultrapure water for 48–72 h, and centrifuged 9000 rpm at 4 °C for 20 min. The supernatant is the soluble regenerated fibroin, which can be preserved at 4 °C for at least one month before becoming irreversible gel. For long-term storage, fibroin solutions should be lyophilized and the resulting powder will be stable for several years at room temperature.

## 3. Fibroin characteristics

### 3.1. Fibroin molecular structure

Fibroin composes of two subunits, a heavy chain (molecular weight (MW) of ∼390 kDa) and a light chain (MW∼26 kDa) linked together via a single disulfide bond at the heavy chain C-terminus (Cys c-20) and the light chain Cys-172. Non-covalently bound to the chains is a glycoprotein called P25 (MW∼25 kDa). The numerical ratio between the heavy chain, the light chain, and P25 is 6:6:1 (Qi et al., 2017). In terms of amino acid composition, *Bombyx mori* fibroin consists mainly of Gly (43%), Ala (30%), and Ser (12%). The heavy chain (45% Gly, 30% Ala, 12% Ser) composes of 12 major hydrophobic domains linking together by 11 minor hydrophilic sections. Each hydrophobic domain contains repetitive sequences of Gly-Ala-Gly-Ala-Gly-Ser and several repeats of Gly-X, with X = Ala, Ser, Thr, Tyr, or Val. The hydrophilic sections have random amino acid sequences. By utilizing intra- and intermolecular hydrogen bonds (mostly between Gly and Ala) and van der Waals forces, the heavy chain can form stable anti-parallel β-sheet crystallites. This property makes fibroin strong, tough, and resistant to water, mild acidity, alkalinity, and other degradation factors (Calvert, 1998; Ki et al., 2009; Keten et al., 2010). On the other hand, the light chain consists of a different proportion of amino acids, 15% Asp, 14% Ala, 11% Gly, 11% Ser, and a trace of cysteine (Kaplan et al., 1993). With non-repetitive amino acid sequences, the light chain is more hydrophilic and has low water resistance, ultimately contributing to the fibroin elasticity (Zhou et al., 2001; Wadbua et al., 2010). Therefore, fibroin is a semi-crystalline structure that has unique properties of both stiffness and strength.

### 3.2. Fibroin polymorphs

Fibroin crystallinity can be altered favorably to yield three distinct polymorphs, silk I, silk II, and silk III. Silk I, found mostly in the silkworm silk glands, has the lowest crystallinity with a dominant number of random coils and α-helices (Pham et al., 2018a). These motifs form a zigzag amorphous structure belonging to the orthorhombic system, which makes silk I thermodynamically unstable and water-soluble (Mottaghitalab et al., 2015; Qi et al., 2017). On the other hand, silk II, found mostly in the silk fibers, has the highest crystallinity with mostly antiparallel β-sheet components, and belongs to the monoclinic crystal system (Pham et al., 2018a). Noticeably, these β-sheet motifs have an asymmetrical structure, which has hydrogen chains of Gly on one side and methyl chains of Ala on the other. The hydrophobic interactions between hydrogen atoms and methyl groups produce a thermodynamically stable silk II. Thus, silk II is stable, has high thermal and mechanical resistances, and water-insoluble. Silk III, found in films formed naturally from the air-water interface of fibroin aqueous solution, is a unique crystalline polymorph of fibroin (Valluzzi et al., 1999). Possesses a threefold helical crystal structure, silk III has crystallinity between silk I and silk II, making it metastable (i.e. less stable than silk II but more than silk I). This conformation is formed due to the fibroin amphiphilic property at the air–water interface. In this polymorph, the Ser residues, which has free hydroxyl groups, line up in the water phase, whereas the Ala residues protrude to the air phase, creating Ser hydrophilic and Ala hydrophobic columns parallel to the fibroin helical axis (Valluzzi et al., 1999).

Interestingly, silk I and silk II are interchangeable by chemical and physical processes, which makes it extremely versatile in the development of biomedical systems from fibroin. In most cases, water-soluble silk I is transformed to insoluble silk II to produce desirable platforms such as scaffolds, films, hydrogels, micro- and NPs. Thus, various methods have been exploited for this reason, and are summarized in Table 1. Among them, three popular methods are based on ionic solutions, organic solvents, and temperature. Ionic solution (i.e. salt) is one of the key factors affecting fibroin crystalline portions, and is dependent upon the particular ions (i.e. chaotropic or kosmotropic), following the Hofmeister series (Hardy et al., 2008). The salting in effect could be observed at low salt concentration, leading to the reduction in fibroin crystallinity. At this concentration, the ionic layer surrounding the fibroin molecules is formed between oppositely charges of salt and fibroin polar amino acid residues. Hence, hydrogen bonds of the crystalline β-sheet become loose, leading to a less ordered structure. Conversely, salting out effect is found at high salt concentration, leading to fibroin precipitation. Organic solvents increase fibroin crystallinity by two mechanisms. First, due to their low dielectric constant, organic solvents reduce fibroin surface charge by dehydration, thus increasing crystalline portions via intra- and intermolecular interactions. Second, they alter the non-covalent interactions of the silk II secondary structures. Temperature also plays a role in fibroin crystallinity. High temperature increases silk II content by decreasing water molecular order (i.e. increasing water entropy), thereby reducing the hydrophobic regions solvation, giving them more chances to form more non-covalent bonds.

**Table 1.** Physicochemical conditions of transforming silk I to silk II.

Conditions	Details	Refs
Ionic solution	Low salt concentration reduces the crystalline β-sheet content, thus, decreases fibroin crystallinity (salting in effect) Antichaotropic (kosmotropic) salts such as potassium phosphate can ‘salt-out’ silk II nanoparticles from silk I solution	(Hardy et al., 2008; Lammel et al., 2010)
Organic solvent	Polar protonic (methanol, ethanol, propanol, isopropanol) and polar aprotonic (tetrahydrofuran, acetone) solvents can induce silk II formation from silk I solution. Although methanol and ethanol are used most frequently, acetone is more effective in making FNPs.	(Zhang et al., 2007)
Temperature	Silk I in aqueous solution gradually and naturally change to silk II gel at room temperature	–
	High temperature (>80 °C) induces silk II formation from silk I film	(Motta et al., 2002)
	At 230 °C, silk I naturally rearranges to silk II	(Pham et al., 2018a)
	Freezing temperature (<–10 °C) induces more β-sheet structures in fibroin nano/microparticles	(Nam & Park, 2001; Cao et al., 2007)
Shear force	The natural process when a silk fiber (silk II) is spun from the silkworm silk glands (silk I). Manual (electro)spinning/stretching or (ultra)sonication of fibroin solution produces similar results	(Jin & Kaplan, 2003; Xie et al., 2006; Pham et al., 2019a)
Crosslinking agent	The addition of the zero crosslinker EDC (1-ethyl-3-(3-dimethylaminopropyl)carbodiimide hydrochloride) increases the silk II polymorph in FNPs	(Pham et al., 2018a)
Polymer	Chitosan increases the fibroin silk II content in the homogenously blended fibroin/chitosan films	(de Moraes et al., 2010; Jeencham et al., 2019)
	Poly(vinyl alcohol) (PVA) induces silk II formation when mixing with fibroin silk I solution	(Wang et al., 2010)
Heavy metal ion	An increase in copper (II) ion (Cu(II)) from 0 to 0.63 mg per g of fibroin solution enhances the silk II conformation. Further increase in Cu(II) reduces the β-sheet fraction	(Zong et al., 2004)
pH	At low pH (<4), silk I solution becomes gel rapidly, which increases the silk II β-sheet content	(Matsumoto et al., 2006)
Enzyme	Protease XIV, although degrades the fibroin, increases its silk II β-sheet amount	(Wongpinyochit et al., 2018)
Water vapor	Post-treatment with water vapor annealing technique increases silk II content in both fibroin microsphere and nanofiber	(Min et al., 2006; Wenk et al., 2008)

On the other hand, the sole purpose of changing silk II to silk I is for making regenerated fibroin from the silk fibers. Hence, only two methods have been proposed, including using strong chaotropic salt solution or enzymes. Practically, the latter is not utilized due to the degradation effect of the enzymes. To break the silk II strong hydrogen bonds, LiBr, CaCl_2_, Ca(NO_3_)_2_ aqueous solution or their mixtures with alcohols are often used (Cheng et al., 2015). Similarly, proteolytic enzymes such as papain decreases the fibroin silk II content (Wongpinyochit et al., 2018).

### 3.3. Fibroin physicochemical properties

The first documented use of silk in biomedical applications was traced back to the first century, when Claudius Galenus of Pergamon treated gladiators with silk fiber as sutures (Holland et al., 2019). However, it was non-sterile silk. Until 1869, Joseph Lister introduced the first sterile silk suture into clinical practice, opening the new era of using silk based materials for healthcare. In 1993, fibroin was recognized by the FDA as a biomaterial (Melke et al., 2016). Fibroin is an ideal biomaterial for NPs formulations, as it possesses potential properties such as (1) water solubility, (2) biocompatibility, (3) biodegradability with nontoxic products, and (4) structure modifiability. Each property, along with factors affecting it, as well as its characterization method (Table 2), are discussed in detail in the following subsections.

**Table 2.** Fibroin characterization methods.

Properties	Characterization methods	Refs
Molecular weight	Gel electrophoresis (i.e. SDS-PAGE)	(Zhang, 2018)
	Non-gel sieving capillary electrophoresis	(Wei et al., 2010)
Degradation/fragmentation	Gel electrophoresis (i.e. SDS-PAGE)	(Zafar et al., 2015)
	Liquid chromatography (i.e. LC–MS/MS)	(Gong et al., 2016)
	Mass loss measurement (i.e. weighing balances)	(Wongpinyochit et al., 2018)
Structure/crystallinity	Fourier-transform infrared spectroscopy (FT-IR)	(Pham et al., 2018a)
	Differential scanning calorimetry (DSC)	(Pham et al., 2018a)
	X-ray diffraction (XRD)	(Pham et al., 2018a)
	Nuclear magnetic resonance (NMR)	(Pham et al., 2018a)
Compositions	Ion exchange chromatography	(Zafar et al., 2015)
	Mass spectrometry	(Lucas et al., 1969)
Solubility	UV–vis spectroscopy	(Pham et al., 2018b)
Mechanical properties	Tensile test	(Jeencham et al., 2019)
Biocompatibility	Cell culture (viability assays)	(Chomchalao et al., 2013)
	Animal model experiments	(Sakabe et al., 1989; Padol et al., 2011; Kanokpanont et al., 2013)

#### 3.3.1. Solubility

Fibroin in its regenerated form possesses high aqueous solubility, which is up to 10% w/v (more than that, the solution becomes extremely viscous and forms gel). This fact is an advantage in FNPs manufacturing as no/less amount of organic solvents is required, comparing to other common materials for NPs such as PLGA. Therefore, unwanted toxicity caused by excess organic solvents to both the human bodily systems and the environment is limited. UV–vis spectroscopy is a simple method to determine the fibroin solubility based on its aqueous concentration at a wavelength of 276 nm (Pham et al., 2018b). Fibroin solubility, which strongly correlates to its crystalline polymorphs, can be controlled favorably by varying experimental factors. As mentioned earlier in section 3.2, silk I amorphous form is more soluble than silk II crystalline form. Thus, methods that increase silk II content will decrease fibroin solubility and vice versa. Techniques to calculate fibroin crystalline fractions have recently been summarized and developed by Pham et al., using Fourier-transform infrared spectroscopy (FT-IR), differential scanning calorimetry (DSC), X-ray diffraction (XRD), and nuclear magnetic resonance (NMR) (Pham et al., 2018a). For example, in the FT-IR spectra, silk II crystalline and silk I amorphous characterized peaks are located at 1622, 1517 cm^−1^, and 1646, 1560 cm^−1^, respectively. By deconvoluting, fitting these peaks and comparing them, fibroin crystallinity can be calculated precisely (Belton et al., 2018; Pham et al., 2018a). Additionally, pH plays a role in fibroin solubility. Fibroin isoelectric point (pI) is 4.53 (3.6–5.2) (Foo et al., 2006), thus, it has negative charge at normal pH of 7.0. A pH value near fibroin pI reduces its charge, resulting in less repulsive force, which decreases its solubility and makes the molecules aggregated. Concentrated acidic solution increases fibroin solubility by the protonation of the amino acid residues of the polymer.

#### 3.3.2. Biocompatibility

As a protein with basic chemical composition, fibroin is a highly biocompatible polymer for clinical use (Cao & Wang, 2009; Melke et al., 2016). First experimental assessed in 1995, fibroin matrix platforms can attach the fibroblast cells and promote their growth significantly (Minoura et al., 1995). The following years, its non-inflammatory property with high biocompatibility to the blood, both *in vitro* and *in vivo*, was also confirmed (Sakabe et al., 1989; Santin et al., 1999). In the US, the amount of commercial biomedical products (i.e. surgical mesh, silk sutures, silk garments) and research patents related to silk fibroin is >100, and this number is increasing dramatically, indicating the fibroin biosafety (Holland et al., 2019). Technically, *in vitro* cell culture and *in vivo* animal models are popularly used to determine fibroin biocompatibility (Table 2). Dependent on the platforms and applications, fibroin might be coated onto the culture container surfaces, followed by cell seeding, or directly treated with the cells. Then, the standard cytotoxicity assays such as mitochondrial activity assays (i.e. MTT, MTS, XTT) or membrane integrity assays (i.e. LDH) can be utilized to access cell viability. For *in vivo* studies, irritation, inflammation, foreign body response, hemolysis, blood clotting, local and systemic toxicity, and pyrogen tests are commonly conducted after the animals being treated with fibroin for a determined amount of time (Sakabe et al., 1989; Padol et al., 2011; Kanokpanont et al., 2013).

#### 3.3.3. Biodegradability

Biodegradability is one of the most remarkable properties of fibroin, leading to the ability to control the characteristics of fibroin-based biomedical platforms. Fibroin in its natural fiber form such as sutures is defined by the United States Pharmacopeia (USP) as a non-degradable material for its negligible tensile strength loss *in vivo* for >60 days. Nevertheless, according to the literature, fibroin degradability is highly dependent on its biomedical platforms, which is mainly due to the effects of proteolytic enzymes such as proteases, and to a lesser extent, hydrolysis (Altman et al., 2003; Farokhi et al., 2014; Wongpinyochit et al., 2018). As a natural protein, fibroin degrades into nontoxic by-product amino acids that can be absorbed, metabolized, and excreted favorably by the body normal functions. Notably, although the amyloid β-fibrils associated with the Alzheimer disease and the fibroin β-sheet degraded products have similar beta structures, these products showed no cytotoxicity to neuronal cells (Numata et al., 2010). Generally, fibroin proteolytic degradation process starts with the hydrophilic segments, including 11 hydrophilic amorphous regions, the C‐terminal, and N‐terminal of the heavy chain, as well as the entire light chain. Then, the degradation of the more crystalline regions follows. The process ends with the most crystalline domain degradation (Holland et al., 2019). To access the fibroin degradation/fragmentation, the most common method is gel electrophoresis (Zafar et al., 2015; Zhang, 2018). Other simpler methods such as mass loss measurement are also utilized (Wongpinyochit et al., 2018). To this end, due to the increase in water solubility of the fragments, the remained intact fibroin platforms lose their weight gradually, which can be determined by washing the platforms, followed by air-drying or lyophilizing, and weighing (Wongpinyochit et al., 2018). To further clarify the fragment sequences or amino acid compositions, gel permeation chromatography and mass spectrometry, which can be coupled with liquid chromatography (LC–MS/MS), can be utilized.

The degradation rates depend on many factors, namely the types of enzymes (i.e. protease XIV, α-chymotrypsin), fibroin crystallinity, biomedical platforms, preparation methods, and the experimental models. For example, papain tends to affect the fibroin crystalline β-sheet regions (Wongpinyochit et al., 2018), whereas α-chymotrypsin cleaves the less-crystalline regions of the protein (Altman et al., 2003). Moreover, silk II has higher crystallinity index than silk I, thus, it takes longer degradation time. In *in vivo* studies, fibroin 3D scaffolds are stable for >1 year when prepared with organic solvents (i.e. more silk II crystalline content), but are completely degraded within 6 months when formulated with aqueous mediums (Wang et al., 2008). On the other hand, the electrospun fibroin scaffolds degrade entirely within 8 weeks (Zhou et al., 2010). Naturally, *Bombyx mori* silkworm produces a trypsin inhibitor in the silk cocoon to protect fibroin from early degradation (Kurioka et al., 1999).

#### 3.3.4. Structure modifiability

The ability to modify, both genetically and chemically, the inherent structure is crucial for a biomaterial as it could increase the material versatility (i.e. produce more functions, enhance specific cell binding and interactions, reduce side effects). To this end, fibroin is a suitable candidate due to its amino acid residues such as Ser, Thr, and Asp, which provide functional groups including hydroxyl and carboxylic that can form covalent and non-covalent bonding with other reagents. Analytical methods such as chromatography and mass spectrometry are commonly used to elucidate the fibroin amino acid composition and sequences.

Recently, Pham et al. successfully utilized PEI, a positively charge polymer, to alter the negatively charged surfaces of FNPs from –20 mV to +30 mV (Pham et al., 2018a). The PEI-functionalized FNPs were proven to be safe to the cells *in vitro*, increased the encapsulated drug entrapment efficiency and loading capacity, as well as enhanced the drug therapeutic efficacy and cellular internalization significantly, compared to the natural FNPs (Pham et al., 2019a). In terms of genetically modification, Teramoto et al. incorporated fibroin with three Met analogs (homopropargylglycine, azidohomoalanine, and homoallylglycine) by adding these analogs to the *Bombyx mori* larvae diet (Teramoto & Kojima, 2015). Furthermore, these amino acids groups can be selectively modified with Cu‐catalyzed azide‐alkyne cycloaddition reactions (click chemistry) because Met residues exist only in the light chain and at the heavy chain N‐terminal. The modified fibroin was also proven to be safe (Teramoto & Kojima, 2015).

## 4. FNPs preparations and characterizations

### 4.1. FNPs preparations

Recently, fibroin has been increasingly utilized in nanopharmaceuticals, based on its appropriate properties for NP formulation. These include high water solubility, biodegradability and biocompatibility, ease of fabrication, freeze-drying favorability, high drug entrapment efficiency and drug loading capacity, controllable particles sizes and release profiles, and modifiable surface (Xie et al., 2006; Wu et al., 2013; Qu et al., 2014; Tian et al., 2014; Li et al., 2016; Gianak et al., 2019; Pham et al., 2019a). Similar to the pure fibroin, FNPs also show excellent safety and biocompatibility in various applications, both *in vitro* and *in vivo* (Kim et al., 2015; Wu et al., 2018; Pham et al., 2019b; Takeuchi et al., 2019). To the best of our knowledge, the earliest FNP formulation was prepared using the desolvation (coacervation, nanoprecipitation) method, reported by Zhang et al. (2007). Since then, many methods have been developed, which can be classified into two categories: the top-down and bottom-up. Each method has its own advantages and disadvantages, and yield FNPs at different sizes, thus, an appropriate selection is crucial in FNPs formulation. These methods are summarized in Table 3.

**Table 3.** Manufacturing techniques for fibroin nanoparticles.

Methods	Details	Loaded drugs	Advantages	Disadvantages	Refs
*Top-down*					
Jet milling	Chopped fibroin is ground in wet attritor milling, followed by spray drying and air jet milling	–	Simple operation Easy to scale up No organic solvent	Wide size distribution Grinding impurities Might reduce silk II content	(Rajkhowa et al., 2009) (0.7–10 µm)
Bead milling	Fibroin small pieces are ground in wet attritor milling, followed by bead milling with pH adjustment	–	Simple operation Easy to scale up Controllable particle size No organic solvent	Grinding impurities Might reduce silk II content Time consuming Chemical residues	(Kazemimostaghim et al., 2013) (0.3–7 µm)
Ball milling	Degummed silk fibers are chopped and ground by planetary ball milling	–	Inexpensive equipment Simple operation Easy to scale up	Wide size distribution Grinding impurities Might reduce silk II content	(Rajkhowa et al., 2008) (0.2–4 µm)
*Bottom-up*					
*Instrumental-based*					
Supercritical fluid	Fibroin solution is atomized with supercritical CO_2_ at controllable high pressure and temperature. FNPs precipitate when CO_2_ evaporates	Curcumin Indocyanine green	Easy to scale up No organic solvent High drug entrapment and loading capacity	Expensive Complicated operation Mainly silk I, need post treatment to induce silk II	(Xie et al., 2017) (28–113 nm) (Chen et al., 2018) (138–194 nm)
Electrospraying	Fibroin solution, forced by the electrical field maintained at high voltage, flows out of a tiny capillary nozzle as small droplets. As water evaporates, FNPs form	Cisplatin	Narrow size distribution Controllable particle size High drug entrapment and loading capacity No organic solvent	Expensive Mainly silk I, need post treatment to induce silk II	(Qu et al., 2014) (59–75 nm)
Spray-freeze-drying	Fibroin solution is sprayed with an ultrasonic nozzle into liquid nitrogen containers. The droplets are then dried in a freeze dryer to form FNPs	Cisplatin	Controllable particle size High drug entrapment and loading capacity No organic solvent	Complicated operation Big particle size (micron) Mainly silk I, need post treatment to induce silk II	(Kim et al., 2015) (3–20 µm)
Laminar jet break-up	Fibroin solution is sprayed and broken-up by a laminar jet, followed by post treatment with methanol or water vapor	Salicylic acid Propranolol Insulin-like growth factor	High drug entrapment Mild condition No organic solvent	Big particle size (micron) Mainly silk I, need post treatment to induce silk II	(Wenk et al., 2008) (100–440 µm)
Microcapillary	Fibroin solution is distributed dropwise on glass slides by a microcapillary. The slides are then freeze-dried and the FNPs are formed	Curcumin	Small and controllable particle size No organic solvent	Complicated operation Difficult to scale up Mainly silk I, need post treatment to induce silk II	(Gupta et al., 2009) (20–130 nm)
Electric field	Conductive electrodes are immersed in fibroin solution for 3 min. At the positive electrode, the formed fibroin gel is then freeze dried to yield FNPs	Bovine serum albumin	Controllable particle size No organic solvent	Mainly silk I, need post treatment to induce silk II	(Huang et al., 2011) (0.2–3 µm)
*Chemical-based*					
Desolvation	Most common method. Fibroin aqueous solution is mixed with a water-miscible organic solvent (i.e. methanol, acetone). The insoluble FNPs formed spontaneously	Alpha mangostin	Easy to scale up Mild conditions Small and controllable size Simple operation	Low drug entrapment and loading capacity Rigorous washing steps Organic solvent residues	(Pham et al., 2019a) (300 nm)
Salting out	Fibroin aqueous solution is mixed with a strong ionic solution (i.e. potassium phosphate). FNPs formed spontaneously	Alcian blue Rhodamine B Crystal violet	Easy to scale up Mild conditions No organic solvent	Difficult to entrap hydrophobic drugs Rigorous washing steps Salt-out agent residues	(Lammel et al., 2010; Mathur & Gupta, 2010) (0.5–2 µm)
Crosslinking reaction	Fibroin solution is mixed with crosslinkers such as EDC to enhance silk II formation, resulting in FNPs	Alpha mangostin	Controllable particle size Simple operation	Rigorous washing steps Crosslinker residues	(Pham et al., 2018a, 2019) (0.3–1 µm)
Reverse microemulsion	Fibroin solution is added into a mixture of surfactant and organic solvent to form microemulsion, which is then broken by alcohol to get FNPs	Rhodamine B	Controllable particle size Simple operation	Surfactant and organic solvent residues Rigorous washing steps	(Myung et al., 2008) (167–169 nm)
Emulsion solvent evaporation	Fibroin solution is mixed with paraffin to form water-in-oil emulsion, followed by water evaporation by heating to yield particles	Bovine serum albumin	Simple operation Mild condition	Big particle size (micron) Organic solvent residues Rigorous washing steps Time consuming	(Srisuwan et al., 2009) (80–150 µm)
Emulsification diffusion	Fibroin solution is homogenized with a water-immiscible organic solvent (i.e. ethyl acetate) to form water‐in‐oil emulsion. By centrifugation, the formed particles are claimed	–	Easy to scale up Simple operation Mild condition	Big particle size (micron) Organic solvent residues Rigorous washing steps Mainly silk I, need post treatment to induce silk II	(Baimark et al., 2010) (100–150 µm)
Polymer blending	Fibroin solution is mixed with a polymeric solution (i.e. PEG, PVA), followed by film/hydrogel forming. FNPs are claimed by dissolving the platforms in water and centrifugation	Bovine serum albumin Dextran Rhodamine B	Simple operation Mild condition No organic solvent Controllable crystallinity and particle size	Polymer residues Mainly silk I, need post treatment to induce silk II	(Wang et al., 2010) (0.3–20 µm) (Wu et al., 2016) (1–100 µm)

Particle size ranges are placed in the parentheses after the references.

Generally, in the top-down methods, gross materials (i.e. silk fiber, lyophilized regenerated fibroin) are size-reduced by mechanical processes such as ball milling to become FNPs. To this end, one can avoid the use of external chemicals (i.e. solvents, enzymes, surfactants) that possibly alter and degrade the fibroin structures. Moreover, the commonly laborious bottom-up preparation processes such as long dialysis time, high costs, lack of safeness due to solvent residues, could be bypassed easily. However, it is difficult to control the FNPs properties, especially the particle morphology, shape, sizes, and polydispersity index. Additionally, challenges in loading or encapsulating the therapeutic entities into the particles hinder the use of the top-down methods. Thus, their applications are limited. In fact, most reports used these methods to prepare blank FNPs for the purposes of cosmetics, filler, and coating. As far as we know, only one research group of Xungai Wang proposed and utilized these top-down methods, including jet-, ball-, and bead-milling, for FNP fabrications (Rajkhowa et al., 2008, 2009; Kazemimostaghim et al., 2013).

On the other hand, the bottom-up methods involve in the assembly of fibroin molecules to form NPs. An increase in the crystalline antiparallel β-sheets results in the condensation of the fibroin molecules, consequently forming particles (Pham et al., 2018a). Therefore, all conditions that can change silk I to silk II polymorph, presented in Table 1, are theoretically practicable. However, not all conditions are suitable for FNP fabrication as some of them might yield other fibroin platforms such as hydrogel, microparticles, or films. The main disadvantages of bottom-up methods are the denaturation of the fibroin structure and the lengthy washing steps after formulation to remove the unreacted reagents. Nevertheless, due to their versatility, bottom-up methods have been extensively investigated, as compared to the top-down methods (Table 3).

Bottom-up methods can be grouped into two categories: instrumental-based and chemical-based. The first group requires the assistance of an industrial machine such as a spray dryer, a freeze dryer, a laminar jet, or an electromagnetic field generator. Thus, they are easy to scale up and are generally utilized in a large batch (pilot scale and higher) FNP preparation. However, the obtained FNPs are mostly in silk I polymorph, hence, it is necessary to induce silk II conformation by further post-treatments with methanol or water vapor. Another disadvantage of these methods is that they require expensive instruments and the need of operational expertise. On the other hand, the chemical-based methods are cheap, easy to operate, especially in small scale, and have mild processing conditions. Nevertheless, they require rigorous washing steps (i.e. dialysis) due to the residual organic solvents, surfactants, and/or polymers. Moreover, they often yield fibroin microparticles rather than NPs because of the difficulty in controlling the precipitating process. Thus, a stabilizer (i.e. a polymer such as PEG) is usually needed to obtain the nanosystems.

### 4.2. FNP sterilization

In clinical applications such as implantation, parenteral and ocular administration, sterility is a crucial issue. Thus, the ability to be sterilized could significantly broaden the versatility of FNPs. This matter is especially important for biomaterials such as fibroin as it can easily attach and support the growth of bacteria and fungi due to its biocompatible surface. Commonly, most FNP formulations possess silk II conformation, which is suitable for sterilization by heat, chemicals, or filtration. Autoclaving is popularly used to sterilize silk II FNPs without major deformation, loss of functions or alteration in particle properties such as size and zeta potential (Wenk et al., 2011), but this might affect the heat-labile encapsulated drugs, namely protein and DNA. Theoretically, chemicals such as 70% ethanol can be used to sterilize FNPs; however, in practice, it is challenging to immerse the particles in these chemicals without any loss or change of encapsulated drugs. In fact, this method is frequently utilized in other fibroin platforms such as scaffolds, implants, or films (Li et al., 2006). Filtration is commonly conducted with a 0.1 or 0.22-µm membrane for most parenteral intravenous and ocular administrations. In case the particle size is larger than 220 nm and the applications are for *in vitro* studies, a 0.45-µm membrane can be used as it eliminates most infectious sources such as bacteria, fungi, yeast, fungal spores, and endotoxins (Pham et al., 2019a).

On the other hand, FNPs with silk I polymorph can only be sterilized by filtration, as other methods might induce silk I-to-silk II formation. Strong irradiation is not suitable for both silk I and silk II. Co(60) gamma irradiation initiates silk I-to-silk II conformational change, with a reduction in gelation time at 37 °C from 10 days to 5 days (Wu et al., 2011), and physicochemically degrades silk II FNPs into smaller MW fragments (Kojthung et al., 2008).

### 4.3. Factors affecting FNP properties

Commonly, all of the mentioned FNP preparation methods in Table 3 can yield fibroin microparticles unintentionally, dependent on the preparation parameters and fibroin MWs. Thus, to adjust the formulating conditions accordingly, it is crucial to characterize the FNPs. Table 4 summaries the common FNP physicochemical properties and their corresponding characterization methods.

**Table 4.** Basic fibroin nanoparticles’ physicochemical properties and their corresponding characterization methods.

Fibroin nanoparticles properties	Characterization methods
Particle size and size distribution	Dynamic light scattering (DLS) (photon correlation spectroscopy, quasi-elastic light scattering)
Zeta potential (surface charge)	Phase analysis light scattering (PALS) Laser Doppler velocimetry coupled with DLS
Particle shape	Transmission electron microscopy (TEM) Scanning electron microscopy (SEM) Atomic force microscopy (AFM)
Surface topology	SEM, AFM
Crystallinity	FT-IR, XRD, DSC, NMR
Drug entrapment efficiency and loading capacity	Drug extraction and purification, followed by UV–vis spectroscopy or liquid chromatography (LC) measurement
Drug aqueous solubility and drug release/dissolution profiles	Drug separation by centrifugation or filtration, followed by UV–Vis spectroscopy or LC measurement
Stability	Physical stability: particle size, size distribution, zeta potential, and shape measurements at each time interval Chemical stability: drug extraction and purification, followed by UV–vis spectroscopy or liquid chromatography (LC) measurement at each time interval

Various factors including fibroin MWs and crystallinity, the encapsulated drug properties, and the preparation conditions, affect FNP properties of particle formation, mean size, size distribution, surface zeta potential, drug encapsulation and release profiles, and stability. Regarding to the particle formation, organic solvents play an important role. Acetone, methanol, and ethanol could induce FNPs from fibroin aqueous solution, whereas acetonitrile only aggregates the fibroin molecules without forming fine particles (Zhang et al., 2007). Polar protonic solvents like methanol, ethanol, propanol, and isopropanol yield globular FNPs, while acetonitrile forms fibroin clumps with no specific shape (Zhang et al., 2007).

In terms of size and size distribution, all mentioned formulating methods yield different particle sizes (Table 3) with relatively narrow size distribution (polydispersity indexes of <0.5), indicating the acceptable qualities of the systems. FNPs prepared by desolvation method with higher initial fibroin concentrations (within 0.25–2.00% w/v) and/or higher ratios between fibroin aqueous solution and ethanol (within 0.33–2.00 v/v), yield significant bigger particle size (within 200–600 nm), and higher polydispersity index (within 0.3–0.5) (Lammel et al., 2010; Pham et al., 2018b). Moreover, our unpublished data demonstrated that high MW fibroin yield bigger FNPs than the low weight one (300 nm vs. 120 nm).

For particle zeta potentials, FNPs normally inherit negative charges at pH 7.0 (i.e. –20 to –30 mV) from their respective fibroin molecular charge. By crosslinking the particles using zero crosslinker EDC, the authors can controllably alter the FNP surface charge from negative to positive (i.e. +30 mV), proportionally to the EDC amount (Pham et al., 2018a). Moreover, by coating the FNP surfaces with positively charge polymers such as PEI or chitosan, the particle zeta potentials are shifted to a more positive value (Wang et al., 2015; Pham et al., 2018a, 2019c).

Concerning the drug encapsulation and release profiles, fibroin crystallinity plays an important role. The methods of altering fibroin crystallinity are reviewed in sections 3.2 and 3.3. To this end, a higher silk II β-sheet content produces tighter and more compact particles, which significantly increase the drug entrapment efficiency, as well as sustain the drug release (Pham et al., 2018a, 2019a). Furthermore, drug loading capacity is found to be dependent on the pKa and solubility of the entrapped drugs. A drug molecule is mostly associated with fibroin via electrostatic interaction, hydrogen bonding, and/or hydrophobic interaction. Therefore, it can decrease fibroin crystallinity by disrupting the hydrogen bonding in the antiparallel β-sheet structures (Pham et al., 2019a). Drug loaded FNP dissolution profiles are strongly correspondent to the investigated media. For example, in a sink condition (i.e. the condition in which the medium can dissolve at least three times the initial total amount of drug), FNPs loading with α-mangostin show a burst release within 30 min. Whereas, in a non-sink condition, which closely simulate the biological system, the same formulations demonstrate a controlled release of over 72 h (Pham et al., 2019a). Furthermore, drugs with high protein binding capacity such as amphotericin B (>95% bound to serum albumin and α_1_-acid glycoprotein) (Bekersky et al., 2002) can also bind strongly with FNPs, consequently lead to no drug release in sink condition (Chomchalao et al., 2020).

Lastly, the storage temperature significantly affects FNP physicochemical stability, even in their lyophilized powder form. Higher temperature (i.e. 25 °C) tends to cause particle aggregation, whereas FNPs are stable to more than 6 months at lower temperature (i.e. 4 °C) due to less inter- and intramolecular interactions (Pham et al., 2019a). The preparation methods have limited effects on FNP stability, instead, the particle surface properties and structures prove noteworthy effects. For instance, using the same desolvation method, non-functionalized FNPs with surface charges of less than ±30 mV tend to aggregate more (i.e. less stable) than PEI-functionalized FNPs or particles with higher charges (Pham et al., 2018a, 2019c).

In summary, these mentioned factors should be greatly considered in the FNP formulation, characterization, and applications, as inappropriate experimental designs might mislead the discussion and the actual particle behaviors.

## 5. Applications

FNPs have been proved to be extremely versatile in delivering a wide range of therapeutic compounds, from small to big molecules, from hydrophobic to hydrophilic substances, and from stable to labile drugs such as proteins, enzymes, vaccines, and DNA. Furthermore, fibroin molecular structure composes of a diverse range of amino acids with functional groups (i.e. amine, hydroxyl, carboxylic, thiol) that can attach different biomolecules or antibodies as markers for active targeting. These functional groups can be altered by, for example, coating with polymers, to control the entrapped drug properties (Pham et al., 2019a). In this review, we separate the FNP applications based on the administration routes, which is closely related to the clinical uses.

### 5.1. Parenteral administration

#### 5.1.1. Small molecule delivery

Despites the fact that most pharmaceutical products in the market are small molecules, they still possess several disadvantages such as hydrophobicity, low permeability, short half-life, nonspecific targeting and distribution, and drug resistance shortly after initial treatment (Mottaghitalab et al., 2015). NPs can effectively overcome these mentioned issues. The NPs with the size ranged 10–200 nm are generally desired to prolong systemic circulation time. NPs of >200 nm are mainly removed via reticuloendothelial system, while NPs of <10 nm might be eliminated by the kidney (Hoshyar et al., 2016). Due to their versatility, FNPs have been increasingly utilized as a delivery system for numerous small molecules. Nevertheless, as mentioned below, most research focus on cancer treatments and anti-inflammation. Therefore, other areas, especially in the chronic illnesses, are wide open and in urgent needed.

Small molecules entrapped in FNPs can be divided into three categories, including chemotherapeutic agents, fluorescence agents for particles tracking purposes, and natural compounds with various therapeutic effects. The first group contains commonly used drugs such as doxorubicin, gemcitabine, floxuridine, paclitaxel, curcumin, methotrexate, emodin, and cisplatin, and has been extensively reviewed elsewhere (Mottaghitalab et al., 2015; Zhao et al., 2015).

The second group composes of various dyes, both hydrophilic and lipophilic, such as rhodamine B (Lammel et al., 2010), alcian blue (Lammel et al., 2010), crystal violet (Lammel et al., 2010), indocyanine green (Chen et al., 2018), and fluorescein isothiocyanate (FITC). Lammel et al. reported the encapsulation of small molecular dyes into FNPs that had their properties dependent on the pH of the salting out solution (Lammel et al., 2010). These particles had a high entrapment efficiency of up to 95% due to the ionic interactions between the positively charged dyes and the negatively charged FNPs. Moreover, the surface charge and FNP crystallinity play an important role in the drug release profiles, as a more positively charged molecule released in a more prolonged trend than the less ones. Similarly, Chen et al. developed indocyanine green loaded FNPs using the supercritical fluid technique (Chen et al., 2018). The particles showed high photothermal stability and pH-responsive, in which the dye released specifically in the tumor acidic environment. Furthermore, these particles can destroy the tumor cells by the light-induced hyperthermia in both *in vitro* and *in vivo* experiments (Chen et al., 2018).

Natural compounds have gained increasingly interests due to their ability to treat various diseases. However, these compounds often possess low aqueous solubility and extensive systemic metabolism, leading to a low therapeutic efficiency. To this end, FNPs prove their ability as a drug delivery system, which successfully incorporate numerous natural compounds such as curcumin (Gou et al., 2019; Xue et al., 2019), quercetin (Lozano-Pérez et al., 2017), alpha mangostin (Pham et al., 2019a), triptolide (Ding et al., 2017), celastrol (Ding et al., 2017), and resveratrol (Lozano-Pérez et al., 2014). In general, all these drug loaded FNPs demonstrated high entrapment efficiency, controllably sustained release profiles, increased drug solubility and stability, decreased drug degradation, reduced drug unwanted toxicity, as well as retained drug therapeutic efficiency, sometimes even enhanced it (Pham et al., 2019b).

For the first time, Pham et al. formulated crosslinked FNPs containing alpha mangostin, a potential chemotherapeutic agent extracted from the mangosteen pericarp, by crosslinking reaction, for anticancer purposes (Pham et al., 2019a). Using EDC or PEI as a crosslinker, these novel FNPs showed spherical shape with a mean size of approximately 300 nm. The particle surface charge was controllable from −15 to +30 mV by varying the type and/or amount of the crosslinkers. Crosslinked FNPs exhibited higher drug entrapment efficiency (70%) and drug loading (7%) than non-crosslinked one. Furthermore, these particles increased the alpha mangostin solubility up to threefold, possessed sustained release of more than 72 h, and reduced the drug hematotoxicity by 90%. Interestingly, on both Caco-2 colorectal and MCF-7 breast adenocarcinoma cell lines, the crosslinked FNPs retained the drug therapeutic effect while exhibited greater cytotoxicity than the free alpha mangostin (Pham et al., 2019a).

Similarly, Ding et al. prepared FNPs loading with triptolide and celastrol, two main compounds in traditional Chinese medicine isolated from thunder god vine, for pancreatic cancer treatment (Ding et al., 2017). These 170-nm negatively charged particles showed a high entrapment efficiency of >80%, a rapid drug release at the lysosomal pH of 4.5, and a delayed release at the plasma pH of 7.4. The drug loaded FNPs demonstrated 2–3-fold more potent (i.e. lower IC50) against MIA PaCa-2 and PANC-1 pancreatic cancer cell lines compared to the free drugs. Moreover, co-treatment with both triptolide and celastrol loaded FNPs synergistically inhibited the cancer cell growth in comparison with each formulation alone (Ding et al., 2017).

Lozano-Pérez et al. reported the encapsulating of the natural antioxidant quercetin into FNPs using desolvation method (Lozano-Pérez et al., 2017). The entrapment efficiency could be achieved of up to 70%, dependent on the quercetin and fibroin ratio. These sustained release particles not only preserved quercetin activity against DPPH but also showed a synergistic scavenging effect due to the intrinsic antioxidant activity of the fibroin (Lozano-Pérez et al., 2017).

The same group also demonstrated the FNP ability to deliver resveratrol, a natural anti-inflammatory compound, in an experimental model of rat colitis (Lozano-Pérez et al., 2014). These 68-nm particles showed non-cytotoxicity to the RAW 264.7 macrophages, as well as demonstrated immunomodulatory properties by promoting the macrophage activity (i.e. nitrite production) in basal conditions and inhibited this activity when stimulated with lipopolysaccharide. Moreover, by evaluating the macroscopic symptoms, inflammatory markers, and intestinal barrier function, *in vivo* study in rat colitis model showed that the resveratrol loaded FNPs had better effect than the pure resveratrol or FNPs alone. This suggested the synergistic effect in anti-inflammatory action of both fibroin and resveratrol (Lozano-Pérez et al., 2014).

#### 5.1.2. Protein delivery

In most cases, therapeutic proteins used in biomedical applications are growth factors and enzymes. Growth factors are polypeptides that can stimulate cell growth and differentiation, which are beneficial in the field of tissue regeneration. Enzymes are a group of proteins responsible for the biological catalytic actions that accelerate chemical reactions, thus, a lack of enzymes might cause severe disorders. Despite the importance of these proteins, their clinical applications often suffer various drawbacks of short half-life, low stability, limited tissue penetration, and potential toxicity. To this end, FNPs have proved their effectiveness in delivering these big molecules to increase protein therapeutic output. Although showing much potentials, most published reports focus on *in vitro* studies, with no further *in vivo* investigation nor clinical trials, halting the possibilities of protein loaded FNPs in the market.

FNPs have been utilized to delivery insulin (Yan et al., 2009; Xue et al., 2017), bovine serum albumin (Chen et al., 2018), vascular endothelial growth factor (VEGF) (Kundu et al., 2010), platelet-derived growth factor (PDGF) (Farokhi et al., 2013), bone morphogenetic protein 2 (BMP-2) (Shi et al., 2013), asparaginase (Zhang et al., 2008), Pin1 isomerase (Kim et al., 2017), protease (Zhu et al., 2011), naringinase (Wu et al., 2013), and β-glucosidase (Cao et al., 2014). Due to its ability to be surface modified and crosslinked, insulin can be conjugated covalently with FNPs at high recovery rate of 90–115%, thus increasing insulin *in vitro* half-life to more than 2.5 times (Yan et al., 2009). On the other hand, albumin, VEGF, PDGF, and Pin1 isomerase can be encapsulated into FNPs or fibroin-based scaffolds, resulting in sustained release, bioadhesive, and enhanced permeation and cellular uptake properties. Furthermore, all of these systems yielded small particle size of less than 300 nm, possessed high entrapment efficiency and loading capacity, and successfully retained the protein efficacy and the enzyme activity. FNPs also have the ability to immobilize the enzymes by chemical interactions between the fibroin tyrosine amino acid residues and the enzyme structures. Consequently, the enzyme stability could be significantly increased while their activities are preserved.

For example, recently, Kim et al. developed FNPs encapsulated in cationic lipid to deliver Pin1 isomerase (a peptidyl prolyl cis–trans isomerase that can binds to the phosphoserine-proline or phosphothreonine-proline motifs of several proteins) directly into the cell cytoplasm (Kim et al., 2017). These FNPs-lipid complexes delivered the enzyme successfully with high efficiency and low cytotoxicity, consequently led to an increase in Runx2 and Smad signaling, followed by the recovery of the osteogenic marker genes expression and the deposition of mineral in Pin1-deficient cells.

#### 5.1.3. Gene delivery

Gene therapy is the method of delivering the required genetic materials such as genes, DNA, or siRNA to the cells to replace, regulate, or express the genes/proteins for cellular functions. This method has shown great potential for the treatments of many diseases, especially the hereditary ones. In most cases, a carrier is needed to overcome several intracellular barriers such as the cell or endosome membranes, as well as to protect the genetic materials from degradation. Due to its versatility, nontoxicity, high transfection efficiency, and DNase resistance ability, fibroin has been investigated as a potential gene delivery system. However, as far as we know, only three publications have been reported recently. Thus, much efforts are necessary in this highly promising field.

Recently, Song et al. fabricated the fibroin-PEI NPs with and without the addition of magnetic NPs, using the salting out method, for the targeted delivery of c-myc antisense oligodeoxynucleotides into the MDA-MB-231 breast cancer cell line (Song et al., 2019). The particle size and zeta potential were controllable by varying the amount of fibroin. Demonstrating less cytotoxicity than the PEI NPs only, both fibroin-PEI NPs, with and without magnetic NPs, could deliver the encapsulated genetic material into MDA-MB-231 cells successfully. By utilizing magnetofection, the fibroin-PEI combined with magnetic NPs showed significantly enhanced uptake into the cells (over 70%) within 20 min, compared to the same carriers via non-magnetofection (Song et al., 2019).

Similarly, *Antheraea pernyi* silk fibroin (another species in the order of Lepidoptera) was used in conjunction with PEI to form the delivery vector for VEGF 165-angiopoietin-1 (Ang-1) dual gene simultaneous expression plasmid (Ma et al., 2014). Due to the fibroin negative charge, the zeta potential of the complex was significantly lower than that of the PEI/pDNA complex (no fibroin). Additionally, the complexes resisted DNase digestion and serum degradation. When transfected into the L929 cells *in vitro*, the cytotoxicity of the fibroin complexes were much lower than the complexes without fibroin. Furthermore, the transfection efficiency and VEGF secretion were increased with the new complexes.

In another study, Shahbazi et al. formulated oligochitosan-fibroin NPs as a delivery system for siRNA therapy, in comparison with the oligochitosan-siRNA polyplexes (no fibroin) (Shahbazi et al., 2015). The particles size was not significantly different compared to the polyplexes (250–450 nm), yet the siRNA loading capacity can be increased by an increase in the fibroin amount. Furthermore, these particles were much more stable in the presence of fetal bovine serum and heparin than the polyplexes without fibroin. Additionally, MTT assays demonstrated that these NPs had lower cytotoxicity as well as demonstrated better gene silencing effect of siRNA, possibly due to the increased loading efficiency, the serum stability and the enhanced cellular uptake (Shahbazi et al., 2015).

#### 5.1.4. Vaccine delivery

Vaccine is a biological preparation, which contains weakened or killed microorganisms, their toxins or surface proteins, that offer immunity to a particular disease. Ironically, the vaccine accessibility in most developing nations is limited by financial costs, despite the desperate needs in public health systems of these countries. Additionally, the vaccine activity and efficacy are heavily dependent on the storage temperature, thus, demand refrigeration throughout the distribution processes. Therefore, it is crucial to investigate alternative systems enabled vaccine delivery that are simple, cheap, easy to manufacture, and thermostable. For this reason, FNPs are potential candidates due to their ability to protect the vaccines from degradation and to be readily taken up by antigen presenting cells. Nevertheless, to the best of our knowledge, a limited number of publications have been reported regarding fibroin, with no papers on FNPs, in vaccine delivery. Thus, this field might be interesting to pay attention in the near future.

Liu et al. formulated and evaluated the immunities of fibroin/chitosan microsphere, using desolvation method, for the delivery of DNA vaccine against infectious bursal disease virus (Liu et al., 2014). The best formula (0.5% chitosan at pH 5.0, 0.6% fibroin, 500 µg/mL plasmid DNA, and 2% Na_2_SO_4_ solution as a crosslinker) produced microsphere with a loading capacity of 89.14% and an average particle size of 1.98 µm. The particles can protect the loading vaccine from DNase I digestion and showed higher immunization activity (ELISA assay) in the serum than the pure vaccine group and the sole chitosan microsphere group (Liu et al., 2014).

### 5.2. Oral administration

Oral administration is the most common route in drug delivery due to its safety profiles, patient compliance, and cost-effectiveness in production. However, conventional formulations such as tablets and capsules, are likely to release the drugs rapidly and uncontrollably. This leads to drug degradation and alteration due to the harsh and unstable environment of the gastrointestinal tract, including the pH variation, the stomach strong acidity, and the presence of digestive proteolytic enzymes and microbiota. Moreover, the common mechanism of drug absorption through gastrointestinal tract is passive diffusion. Consequently, most of the initial dose is not absorbed but is metabolized and excreted. FNPs possess favorable characteristics to overcome all mentioned issues and become interest candidates for oral administration of therapeutic compounds. First, due to its mucoadhesive ability, FNPs can adhere tightly to the gastrointestinal mucosa or intestinal epithelial cells (Peyer’s lymphatic M cells), followed by cellular internalization via endocytosis. Thus, the encapsulated drugs can enter the blood stream effectively and intactly. Generally, particles with smaller size (<200 nm) resulted in higher cellular internalization and better transportation through intestinal cell layers than the bigger particles (>500 nm) (Banerjee et al., 2016). Its small particle size also enhances the drug solubility, which can improve the intestinal absorption via diffusion. Second, although being degraded by intestinal proteolytic enzymes such as α-chymotrypsin, the FNP degradation rate is usually slow to days, which gives enough time for the particles to do their job (gastrointestinal transit time is <24 h, not counting the colon residence time). Finally, as mentioned earlier, fibroin pI is 4.53, thus, FNPs are generally colloidal stable at a pH ≥ 5 (i.e. pH range of 5–14) due to the repulsive forces generated from their negatively surface charge. Moreover, research has shown that even at pH < 5, fibroin at low concentration (0.2% w/w) might not be aggregated because of the steric hindrance effect (Jia et al., 2014). Thus, FNPs are stable in a wide range of pH, making them suitable for oral delivery. Novel approach in this field is the combination of FNPs and conventional dosage forms such as capsules, which give further protection in the harsh oral conditions, and control the drug release in targeting areas more precisely.

Gou et al. developed curcumin loaded FNPs functionalized with chondroitin sulfate as a multi-bioresponsive platform for the treatment of ulcerative colitis, using both oral and intravenous administrations (Gou et al., 2019). These spherical particles had an average size of 175.4 nm, a narrow size distribution, and a negative surface charge of −35.5 mV. The particle surface coated with chondroitin sulfate yielded notably targeted drug delivery to macrophages, consequently enhanced the anti-inflammatory activities of curcumin. Upon endocytosed by the macrophages, these NPs can be favorably triggered, followed by intracellular drug release, with the intrinsic stimuli such as acidity, glutathione, and reactive oxygen species. Moreover, *in vivo* animal experiments in mice demonstrated that these NPs, through both oral and intravenous administrative routes, could reduce the ulcerative colitis symptoms effectively, while maintained the intestinal microbiota integrity and improve the mice survival rate (Gou et al., 2019).

In another *in vivo* study, curcumin loaded FNPs were also utilized for the investigation of the relationship between particle size and the drug pharmacokinetic profiles (Wu et al., 2018). By using the polymer blending method with PEG 10,000 at various fibroin concentrations, the particle size ranging from 229 to 2286 nm, and the entrapment efficiencies from 22 to 41%, proportionally to the fibroin amount, were obtained. Furthermore, curcumin was released slower from the larger particles than the smaller ones. Using HPLC–MS/MS method, the authors determined the curcumin pharmacokinetic parameters in rat plasma after a single dose orally administration, in comparison with the pure curcumin. After 24 h, the 800-nm FNPs showed longer plasma circulation time (*T*_max_=1.76 ± 0.10 h) than the smaller FNPs (size = 200 nm, *T*_max_=1.17 ± 0.07 h). Conversely, the *C*_max_ of 200-nm FNPs was twofold greater than that of the 800-nm FNPs (446.43 ± 142.18 vs. 228.05 ± 46.73 ng/mL). Remarkably, the 200-nm FNPs increased curcumin bioavailability up to 17 times higher than the pure drug, as well as doubled its half-life from 0.89 ± 0.21 h to 1.51 ± 0.23 h (Wu et al., 2018).

Roy et al. formulated FNPs from the *Samia cynthia ricini* silkworm (another species in the Lepidoptera order) using the bead milling method to deliver anticancer milk proteins bovine lactoferrin for the purpose of breast cancer treatment (Roy et al., 2016). The authors’ main aim was to investigate the molecular mechanisms underlying the interactions between the FNPs and breast cancer cell lines. Nevertheless, for the determination of FNPs and the drug degradability by the digestive enzymes in gastrointestinal tract, their *ex vivo* loop assay proved some useful information for oral administration of FNPs. The results suggested that after 6 h from the experiment onset, these 200–300 nm particles were co-localized, in intact form, mainly in the serosa, mucosal layers, and sub-mucosal layers of the ileum, as well as in the intestinal gland, crypt, and plicae circulares portion, consequently leading to an enhanced uptake and absorption of the FNPs through the ileum (Roy et al., 2016). Thus, FNPs can be utilized to transport labile proteins through the intestinal tract as an oral drug delivery system, without the loss of the drug functions nor the carrier intactness.

### 5.3. Transdermal administration

Skin is the largest organ of the body, with its outermost layer, stratum corneum, works as a barrier against external factors such as bacteria, viruses, and chemical agents. Therefore, for transdermal administration, the therapeutic agents need to permeate through this barrier to reach deeper layers (i.e. dermis) and further to the systemic circulation. Generally, NPs with a mean size of ≤4 nm can penetrate and permeate the intact skin, the size of 4–20 nm might permeate intact and damaged skin, while the size of 21–45 nm can only penetrate and permeate the damaged skin, and with a size of >45 nm, NPs cannot penetrate nor permeate the skin, but distribute in the stratum corneum (Filon et al., 2015). To this end, fibroin proves itself an interesting carrier due to the amphiphilic properties of both crystalline hydrophobic regions and amorphous hydrophilic domains, as well as its ability to control the particle size favorably. Although proving much potentials, research in this area is limited. Hence, it might be an interesting approach for widening the FNP transdermal application in the near future.

In a study conducted by Takeuchi et al., spherical FNPs with a mean size of 42.3 nm and a narrow size distribution with polydispersity indexes of less than 0.3 were successfully prepared using desolvation method (Takeuchi et al., 2019). The particles were stable in the liquid dispersion form for a week. FNPs were then conjugated with fluorescent NHS-rhodamine for particle tracking. Skin permeability *in vivo* test was evaluated using mice. Six hours after administration, fluorescent signals were found not only in the stratum corneum, but also in the hair follicles, the epidermis, and the dermis layers, indicating that small size FNPs can penetrate the stratum corneum via paracellular route and proceed deeply into the skin (Takeuchi et al., 2019).

In another study, Mao et al. reported that fibroin, in its hydrogel form, can enhance the transdermal delivery of the incorporated curcumin loaded polymeric NPs in psoriatic mouse model (Mao et al., 2017). Curcumin is a highly lipophilic compound that is easily trapped in the stratum corneum. Thus, the authors encapsulated curcumin in a self-assembly amphiphilic polymer named RRR-α-tocopheryl succinate-grafted-ε-polylysine. The spherical 24.4-nm particles, with a drug loading capacity of 3.49% and an entrapment efficiency of 78.45%, were then further incorporating in fibroin hydrogel. By the formation of a thin fibroin gel layer, occlusive effect occurred, which kept the skin surface hydrated and increased the formulation contact time to the skin, consequently translocated the NPs across the deeper skin layers (Mao et al., 2017).

### 5.4. Ocular administration

Drug delivery locally to the eyes for ocular diseases has remained one of the most challenges in pharmaceutical sciences. The eyes compose of various barriers, namely the ocular surface epithelium, the tear film, the blood–aqueous, and the blood–retina barriers, as well as the rapidly fluid clearance from the choroidal, conjunctival, and lymphatic vessels. In the market, >90% of all topical ophthalmic formulations are eye drops (Gulsen & Chauhan, 2005). Unfortunately, only about 5% of the total dose in the eye drops reaches the intraocular tissues and yield therapeutic effects, the remaining 95% is lost due to tear drainage into the nasolacrimal duct, which further empties into the nasal cavity, and ultimately absorbed into the bloodstream (Lang, 1995). This does not only reduce the drug efficacy, but also causes undesirable systemic side effects. Additionally, the extremely short residence time of about 2 min increases the eye drop administration frequency and reduces patient compliance (Gulsen & Chauhan, 2005). To this end, NPs can overcome all mentioned problems with their ability to control the particle sizes and the drug release in the ocular surfaces. For example, after intravitreal administration, micron-sized particles (i.e. 2 µm) mostly stay in the vitreous cavity, the 200-nm NPs distribute in the vitreous cavity and the inner limiting membrane, whereas the 50-nm NPs remain in the retina for at least 2 months (Jiang et al., 2018). Similarly, for topical applications, micron-sized particles could not diffuse into the sclera, whereas NPs of <100 nm are transported successfully to the sclera, with higher amount for smaller NPs (73 nm) compared to larger ones (94 nm) (Joseph & Venkatraman, 2017). In fact, fibroin has been long used for ocular diseases; however, most applications are based on films and hydrogels as a delivery platform (Tran et al., 2018). As far as we know, a limited number of publications related to FNPs for the treatment of the eyes have been reported. Possessing ideal properties, FNPs are emerging systems for the new era of ocular drug delivery.

Chomchalao et al. successfully developed amphotericin B loaded FNPs, with the combination with the polymer PEG 400, as a ready-to-use eye drops for fungal keratitis treatment (Chomchalao et al., 2020). The particles prepared by desolvation method exhibited homogeneous spherical particles with a mean size of ∼270 nm, the zeta potential of ∼–17 mV, and an entrapment efficiency of ∼65%. Amphotericin B was tightly entrapped in FNPs in amorphous molecular dispersion and monomeric form, with no detectable drug release in sink condition after 6 h. Nevertheless, the drug loaded FNPs retained the antifungal activity against *Candida albicans*, and showed less cytotoxicity to human corneal epithelial cell line as compared to the marketed amphotericin B deoxycholate (Chomchalao et al., 2020).

Yang et al. formulated the FNPs using desolvation method, encapsulating the bovine serum albumin tagged with FITC for intravitreal injection to the retina (Yang et al., 2019). The FNPs had a narrow size distribution with a mean particle size of 179.1 ± 3.7 nm and a zeta potential of > −25 mV, with no observable *in vitro* cytotoxicity in human retinal pigment epithelial AREP-19 cells. Interestingly, compared to the free drug, FNPs distributed locally and extended retention time in the retina *in vivo* by intravitreal injection in New Zealand white rabbits (Yang et al., 2019).

Similarly, FNPs loaded with bovine serum albumin were also investigated by the same research group for transscleral administration under ultrasound exposure (Huang et al., 2014). The ultrasound at 1 MHz, 0.5 W/cm^2^, 5 min continuous wave, significantly improved the penetration efficiency of FNPs as compared with the free drug passive pathway, while caused no damages to the ocular tissue of the rabbit isolated sclera. FNPs can adhere rapidly and strongly to the outer scleral tissues, followed by migration into the interior up to one week after treatment (Huang et al., 2014).

In an *in vitro* study, Dong et al. showed that the fibroin coated liposomes containing ibuprofen can sustain release the encapsulated drug and increase the drug corneal adhesion and permeation on human corneal epithelial cells, as compared to the free drug and the uncoated liposomes (Dong et al., 2015). Ibuprofen can enter the cells as rapidly as after 7 min of exposure, and the drug concentration increased gradually up to 12 h, with no detectable cytotoxicity.

### 5.5. Orthopedic administration

Due to the extensive use of orthopedic devices and joint replacements, severe bone and joint infections have been increased significantly, which possess various impacts on patients. Current osteomyelitis treatments, including parenteral antibiotics, hyperbaric oxygen, and surgery are not sufficient. Furthermore, in the disease advanced stages, due to the shortage in blood supply, bone necrosis restricts the intravenous delivery of antibiotics to the infection site. Additionally, the short half-life of antibiotics and their systemic toxicity limit the application of high doses of drugs. Therefore, the use of local treatment is favorable. Recently, FNPs have gained much interest in this research field due to their nontoxic, biodegradable, as well as the mucoadhesiveness, which can enhance the particles retention time at the infectious area. Particle size plays an important role in the interactions between NPs and bone cells. However, there is controversy in this issue, as one study stated that NP sizes of up to 40 nm decreased osteoblastic cell proliferation and viability, yet in another study, tiny NP size of 20 nm enhanced osteoblast-like cell growth (Tautzenberger et al., 2012). Due to the necessity in prolonging the drug release to weeks or months, FNPs are usually incorporated into another scaffold such as hydrogel. This makes the preparation process more complex and hinders the scaling-up process. Hence, further research is required to overcome this issue.

Besheli et al. successfully incorporated the antibiotic vancomycin into FNPs using desolvation method, followed by complexation with the fibroin scaffolds, for the local bone infection treatment (Hassani Besheli et al., 2017). With a maximum drug loading capacity of 18.84% and an entrapment efficiency of >90%, these scaffolds can sustain release vancomycin to more than 2 weeks and the release rate at pH 4.5 (i.e. at the infectious site due to microbial anaerobic metabolism) was slower than at pH 7.4 (i.e. in the systemic circulation). In an *in vitro* disk diffusion test, the scaffolds retained the antibacterial activity of vancomycin against methicillin-resistant *Staphylococcus aureus* (MRSA), the main pathogen causing osteomyelitis. Furthermore, in *in vivo* test in rat bone infection model, the local implant FNP scaffolds at the infectious site demonstrated significant better outcomes than the other treatment groups after 6 weeks of implantation, analyzed by radiographic and histopathological analysis (Hassani Besheli et al., 2017).

In another study, the growth factor VEGF was successfully loaded in the spherical 97-nm FNPs, followed by incorporated in a fibroin scaffold loaded with vancomycin, for the bone infection treatment (Hassani Besheli et al., 2018). The scaffold sustainably released both vancomycin and VEGF up to 21 days, while preserved the drug bioactivity for over 28 days, thus, both induced osteoblasts proliferation and removed infection (Hassani Besheli et al., 2018).

### 5.6. Respiratory administration

The lung is a potential target for drug delivery for both local and systemic treatments. Locally, one can treat lung and respiratory diseases (i.e. lung cancer, tuberculosis) with a reduced dose and side effects as compared to the conventional dosage forms. Systemically, due to the lung large surface area, drug can be absorbed rapidly and effectively without being degraded by the first-pass metabolism as in oral administration. To effectively deliver the FNPs to the lung, their aerodynamic particle size proves an important factor. For spherical particles, the aerodynamic diameter is dependent on the particle density and geometric diameter (i.e. diameter measured by DLS or SEM/TEM techniques). Particles with aerodynamic diameters of (1) >10 µm, are accumulated in the oropharyngeal region, mostly the larynx; (2) from 5 to 10 µm, are mostly stuck in the large airways; (3) from 1 to 5 μm, can be deposited in the small airways and alveoli; and (4) <500 nm, might be diffused back to the air via exhalation (Labiris & Dolovich, 2003; Kim et al., 2015). Various inhalation products designed to locally treat the lung diseases are undergoing clinical development, which make FNPs an interesting candidate for this purpose. This research area is relatively innovative and remains wide open, and thus, demands great efforts in the near future.

Kim et al., for the first time, formulated cisplatin loaded FNPs as a pulmonary drug delivery system for targeted lung cancer treatment, using spray-freeze-drying method (Kim et al., 2015). Mannitol was used as an excipient to compress the FNP powder into the dry powder inhaler equipment by an *in vitro* aerosolization impactor. All particles demonstrate high aerosolization performance measured by *in vitro* lung deposition, which was comparable to the commercial dry powder inhalers. Moreover, the cisplatin loaded FNPs showed high cytotoxicity to the A549 human lung epithelial cell line, whereas the blank FNPs were biocompatible. Interestingly, the blank FNPs improved the cell migration in wound healing experiment, while the drug loaded particles suppressed the cell growth and movement significantly (Kim et al., 2015).

## 6. Challenges, conclusions, and outlooks

Although possessing numerous advantages as a drug delivery system utilized in many administrative routes, fibroin still has some disadvantages needed to be overcome. First, the sericin removal process from silk fibers has to be completed and adequate because sericin might cause immunogenic actions. Second, the slow degradation of silk II crystalline antiparallel β-sheet domains might be a drawback in some applications that need to eliminate the nanoparticulate carrier rapidly and entirely. Silk II content can be measured and calculated using analytical methods such as FT-IR, XRD, DSC, and NMR, thus, a careful consideration and calculation might help in choosing the right preparation methods, as each method yields different crystalline amounts. Third, as a protein, fibroin might get proteolytic attack by the immune system such as the macrophages and giant cells, consequently leads to off-target drug release due to the encapsulation and formation of granuloma inside these cells (Wang et al., 2008). Coating/incorporating fibroin or FNPs with PEG or other hydrophilic polymers might solve the problems. Fourth, similar to other natural products, fibroin can be extracted from various sources, thus, the properties of each batch are slightly different owing to the post-translational process variations between both species and individuals. Consequently, a standardized extraction method and the sample properties (i.e. MW) are necessary. The use of genetically recombinant fibroin could overcome this issue. Finally, although proving much potential in protecting the encapsulated drugs, increasing their stability and prolonging their release profiles, FNPs are not the targeted drug delivery systems on their own. Therefore, low therapeutic efficiency and systemic toxicity might occur because of the unspecific targeting. To this end, fibroin surface modification with specific ligand (i.e. folic acid for tumor targeting) via both covalent and non-covalent bonding proves it effectiveness.

Nevertheless, these aforementioned limitations and challenges could be overcome in one way or another. Therefore, fibroin, especially FNPs, has a great tendency to be a delivery system of choice for various therapeutic agents including small molecule drugs, protein drugs, genes, and vaccines. Furthermore, numerous FNP administration routes have been investigated such as parenteral, oral, transdermal, ocular, local bone implantation, and respiratory. Due to the FNP favorable properties, further studies should be focused on the less investigated yet potential routes, namely ocular and respiratory. Finally, yet importantly, most studies on FNPs are based *in vitro* and *in vivo* experiments, thus, more clinical trials should be conducted to potentially translate the use of FNPs to the market.
